# Association between visitation restriction during the COVID-19 pandemic and delirium incidence among emergency admission patients: a single-center retrospective observational cohort study in Japan

**DOI:** 10.1186/s40560-020-00511-x

**Published:** 2020-12-07

**Authors:** Kenji Kandori, Yohei Okada, Wataru Ishii, Hiromichi Narumiya, Yoshiro Maebayashi, Ryoji Iizuka

**Affiliations:** 1 Department of Emergency and Critical Care Medicine, Japanese Red Cross Society, Kyoto Daini Hospital, 355-5 Haruobicho Kamigyoku, Kyoto, 602-8026 Japan; 2 Preventive Services, School of Public Health, Kyoto University, Kyoto, Japan; 3 Department of Primary Care and Emergency Medicine, Graduate School of Medicine, Kyoto University, Kyoto, Japan; 4 Department of Psychiatry, Japanese Red Cross Society, Kyoto Daini Hospital, Kyoto, Japan

**Keywords:** ABCDEF bundle, COVID-19, Delirium, Emergency department, Family, Visitation restriction

## Abstract

**Aim:**

This study aimed to identify the association between total visitation restriction because of the coronavirus 2019 (COVID-19) pandemic and the incidence of delirium for emergency inpatients.

**Methods:**

This was a single-center, retrospective, observational cohort study conducted at a tertiary critical care center in urban Kyoto, Japan. Adult emergency patients hospitalized between January 1, 2019, and June 30, 2020, were recruited. In response to the COVID-19 pandemic, the authors’ hospital began restricted visitation on March 28, 2020. This study defined *before visitation restriction* as January 1, 2019, through March 31, 2020, and *after visitation restriction* as April 1, 2020, through June 30, 2020. We did not restrict emergency services, and there were no changes in the hospital’s routine, except for visitation restrictions. The primary outcome was the incidence of delirium. The adjusted odds ratio (AOR) with 95% confidence interval (CI) for delirium incidence was calculated to compare the before and after visitation restriction periods, and the logistic model was used to adjust for seven variables: age, sex, ward type on admission, primary diagnosis, ventilator management, general anesthesia surgery, and dementia.

**Results:**

Study participants were 6264 patients, median age 74 years (56–83), and 3303 men (52.7%). The total delirium incidence in entire research period was 2.5% (158 of 6264 patients), comprising 1.8% (95/5251) before visitation restriction and 6.2% (63/1013) after visitation restriction. The AOR for delirium incidence was 3.79 (95% CI, 2.70–5.31) after visitation restriction versus before visitation restriction. Subgroup analysis showed no apparent interaction for delirium incidence.

**Conclusion:**

Visitation restriction was associated with an increased incidence of delirium in emergency inpatients.

## Background

The incidence of coronavirus disease (COVID-19) infections continues to increase worldwide. In Japan, a national state of emergency was declared on April 16, 2020, which was then lifted on May 25, 2020, once the incidence was thought to have declined to a targeted plateau; however, now the number of infections is again increasing.

Prevention of transmission of the COVID-19 infection within the hospital setting is important to maintain the function of the hospital, and thus various measures have been adopted to meet this goal. In particular, many hospitals have restricted visits to inpatients by their family members and close contacts. The hypothesis in this study is that visitation restrictions to inpatients may increase their risk of delirium. *Delirium* is defined as a form of acute brain dysfunction that often occurs in acute care settings [1] and is associated with many adverse outcomes after hospitalization or discharge, including increased mortality [2–14]. Prevention of the development of delirium is important, and family engagement is accordingly incorporated as an “F” component in the ABCDEF bundle, which describes the following strategy: *assess*, prevent, and manage pain; *both* spontaneous awakening trials and spontaneous breathing trials; *choice* of analgesia and sedation; *delirium*: assess, prevent, and manage; *early* mobility and exercise, and *family* engagement and empowerment [15]. Therefore, visitations between patients and their families are important to prevent the delirium [15, 16].

Despite the clinical importance of delirium in this patient setting, no reports to date have addressed the incidence of delirium under visitation restrictions in response to the COVID-19 pandemic. In context of this pandemic and the unprecedentedly large scale of visitation restriction, the purpose of this study was to identify the association between visitation restriction and the incidence of delirium in emergency inpatients.

## Methods

### Study design

This study is a single-center, retrospective, observational, cohort design. The study was approved by the Clinical Research Ethics Committee of the Japanese Red Cross Society Kyoto Daini Hospital (Approval ID Sp2020–7). The ethics committee waived the requirement for informed consent because of the anonymous nature of the data.

### Setting

The study was performed at the Japanese Red Cross Society Kyoto Daini Hospital in Kyoto City, Japan, which is an urban area with a population of approximately 1.5 million. The total number of ambulance calls is approximately 90,000 cases annually for the entire city [17]. The authors’ 672-bed hospital is one of the four critical care medical centers in Kyoto City and is located at the center of the city. Generally, critical care medical centers are certified by the Japanese Ministry of Health, Labour and Welfare; can accept emergency and severely ill patients transported by ambulance, including cardiac arrest, trauma, stroke, and sepsis patients; and can provide the specialized treatment in an intensive care unit, stroke care unit, and high care unit. In 2019, the total number of emergency department cases was 7610 patients who arrived by ambulance and 20,769 patients who were “walk-in” status, arriving by other means.

### Study population

This study included all adult (age ≥ 18 years) patients hospitalized for any causes via our emergency department between January 1, 2019, and June 30, 2020.

### Visitation restriction

In response to the COVID-19 pandemic in Japan, the study hospital changed the visitation policy to restrict visits beginning on March 28, 2020. The visit restrictions meant that the patient’s family and other close contracts were not allowed to visit the hospital ward or to have contact with inpatients in principle even for short periods of time. The study intervention periods were defined as *before visitation restriction*, from January 1, 2019, through March 31, 2020, and *after visitation restriction*, April 1, 2020, through June 30, 2020. We have been accepting inpatients because of COVID-19 since April, 2020, but this has not significantly affected our normal practice and has not restricted our emergency services. Even for inpatients, there were no changes in the hospital’s routine, except for visitation restrictions.

### Data collection

Clinical data were obtained by an electronic chart review and the Japanese Diagnosis Procedure Combination (DPC) database of the Japanese Red Cross Society Kyoto Daini Hospital. These clinical data were collected through the electronic chart review: date of admission and discharge, patient age, patient sex, ward type on admission, and psychiatrists’ medical record about delirium. The DPC database [18] includes administrative claims and discharge abstract data for all inpatients. The following information for each patient was recorded in the DPC using a uniform data submission form: age; sex; state of consciousness on admission/discharge; activities of daily living on admission/discharge; primary diagnosis; comorbidities, including dementia, on admission; post-admission complications coded using the International Classification of Diseases, 10th Revision; medical procedures, including ventilator management and general anesthesia surgery; and discharge status. Patients were categorized by age as 18–64 years, 65–74 years, and ≥ 75 years, and by ward type on admission as emergency ward and general ward; the *emergency ward* was defined as the intensive care unit, stroke care unit, and high care unit. Patients were categorized as having ventilator management if they required > 5 h of ventilator management.

### Outcome

The primary outcome was the incidence of delirium. In this hospital, the psychiatry department provides consultation as needed for the inpatients who are hospitalized in other departments, including their psychiatric diagnosis and intervention. *Delirium* in this study was defined as receiving a diagnosis of delirium by the psychiatrists and requiring their intervention for delirium during hospitalization. Although delirium is challenging to diagnose and is a diagnosis that tends to be missed [2], this study focused on diagnostic accuracy and clinically problematic delirium by limiting the incidence of delirium to that which required intervention by psychiatrists. The psychiatry department diagnosed delirium according to the 5th edition of American Psychiatric Association’s Diagnostic and Statistical Manual of Mental Disorders (DSM-5) [19], which is the current reference standard diagnostic criteria [20]. The policy in psychiatric department to diagnose and manage the delirium did not change over the study intervention periods of before and after visitation restriction.

### Variables selection

Based on previous studies [1, 20–26], as potential confounding factors to assess the association between incidence of delirium and visitation restriction, the study used these seven variables: patient age, patient sex, ward type on admission, primary diagnosis, ventilator management, general anesthesia surgery, and dementia.

### Sample size estimation

We estimated that at least 100–120 cases of delirium would be required to account for the confounders using a logistic model based on the generally accepted rule of 10 events per variable [27]. Considering this viewpoint, we assumed that including cases from January 2019 to March 2020 before restriction would result in an adequate sample size to perform the analysis.

### Statistical analysis

Data for patient characteristics were described as a median with an interquartile range (IQR) for continuous variables and as a number with percent for categorical variables. The crude and adjusted odds ratios (AORs) of delirium incidence with 95% confidence intervals (CIs) were identified using the multivariable logistic model including all confounders. Furthermore, subgroup analysis was conducted to evaluate the interaction in the association between visitation restriction and delirium incidence. Crude odds ratios with 95% CIs were using univariable logistic regression models. In addition, *P* values for interaction were calculated, and the interaction was evaluated [28].

Moreover, we performed a sensitivity analysis that also accounted for physiological severity as a covariate to demonstrate the robustness of the primary analysis because of the idea that patient severity might also be a potential confounder. However, we could not obtain the Sequential Organ Failure Assessment (SOFA) score for all patients. Therefore, we alternatively evaluated the physiological status as “assumed SOFA score” using the following factors: level of consciousness, ventilation management, catecholamine usage, renal replacement therapy, and plasma exchange (see the detail in Additional file 1). As in the sensitivity analysis, we added “the assumed SOFA score” as a covariate and performed logistic analysis similar to the primary analysis.

All *P* value analyses were two-sided, and *P* < 0.05 was considered significant. Missing data were not replaced or estimated. Statistical analyses were performed using JMP Pro 14 software (SAS Institute, Cary, NC, USA).

## Results

### Patient characteristics

**Fig. 1:**
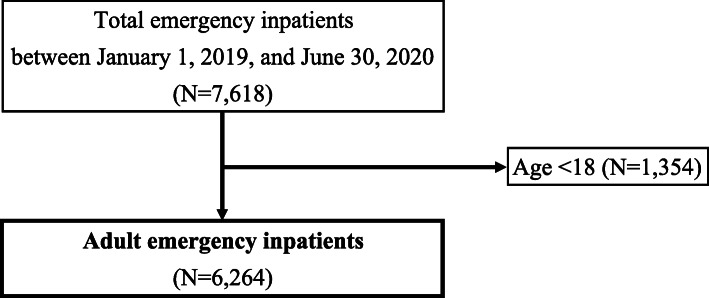
Flowchart of the study population

Of the total 7618 emergency patients admitted to the study hospital through our emergency department between January 1, 2019, and June 30, 2020, on the basis of age < 18 years, 1354 patients were excluded. The remaining 6264 patients were included for analysis (Fig. 1). Patient characteristics are shown in Table 1. In summary, median age was 74 years (IQR, 56–83), and 3303 (52.7%) patients were male. Baseline characteristics were similar between the two cohorts. Detailed primary diagnosis is described in Supplementary Table 2.

**Table 1:** Patient characteristics before and after visitation restriction

Variables, number, (% or IQR)	All patients (***N*** = 6264)	Visitation restriction	
		Before (January 2019–March 2020) (***N*** = 5251)	After (April 2020–June 2020) (***N*** = 1013)
**Age, years**	74 [56–83]	74 [56–83]	75 [58–84]
**Age group,** ***n***			
18–64 years	2059 (32.9%)	1732 (33.0%)	327 (32.3%)
65–74 years	1094 (17.5%)	929 (17.7%)	165 (16.3%)
≥ 75 years	3111 (49.7%)	2590 (49.3%)	521 (51.4%)
**Sex, male,** ***n***	3303 (52.7%)	2762 (52.6%)	541 (53.4%)
**Primary diagnosis,** ***n***			
Neurological disease	1243 (19.8%)	1038 (19.8%)	205 (20.2%)
Cardiovascular disease	794 (12.7%)	679 (12.9%)	115 (11.4%)
Respiratory disease	580 (9.3%)	480 (9.1%)	100 (9.9%)
COVID-19 infection	10 (0.2%)	0 (0.0%)	10 (1.0%)
Digestive disease	1249 (19.9%)	1040 (19.8%)	209 (20.6%)
Pregnancy, gynecological disease	413 (6.6%)	365 (7%)	48 (4.7%)
Trauma	1042 (16.6%)	876 (16.7%)	166 (16.4%)
Others	943 (15.1%)	773 (14.7%)	170 (16.8%)
**Ward type on admission,** ***n***			
Emergency ward	3089 (49.3%)	2615 (49.8%)	474 (46.8%)
General ward	3175 (50.7%)	2636 (50.2%)	539 (53.2%)
**Ventilator management,** ***n***	615 (9.8%)	518 (9.9%)	97 (9.6%)
**General anesthesia surgery,** ***n***	950 (15.2%)	783 (14.9%)	167 (16.5%)
**Dementia,** ***n***	1161 (18.5%)	957 (18.2%)	204 (20.1%)
**Death in hospital,** ***n***	431 (6.9%)	368 (7.0%)	63 (6.2%)

Values are median (interquartile range [IQR]) or number (percentage)

### Outcome

**Fig. 2:**
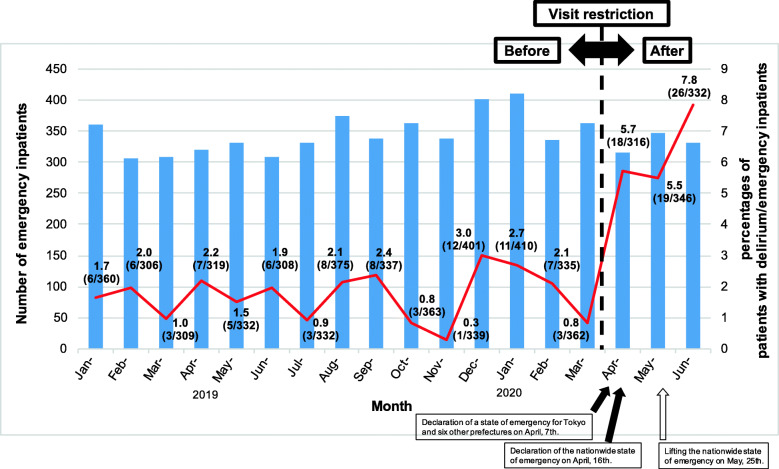
Trends in the incidence of delirium in emergency inpatients. The bar graph shows the number of emergency inpatients. The line chart shows the percentage of patients with delirium/emergency inpatients per month. The study hospital enforced visitation restrictions beginning from March 28, 2020. An increase in the incidence of delirium was observed after the implementation of the visitation restriction

The monthly incidence of delirium and the total number of emergency admissions are shown in Fig. 2. The total incidence of delirium in entire research period was 2.5% (158/6264 patients). During the entire before visitation restriction period (January 2019–March 2020), the delirium incidence was 1.8% (95/5251) compared with a delirium incidence of 6.2% (63/1013) for the after visitation restriction period (April–June 2020).

### Primary analysis

**Fig. 3:**
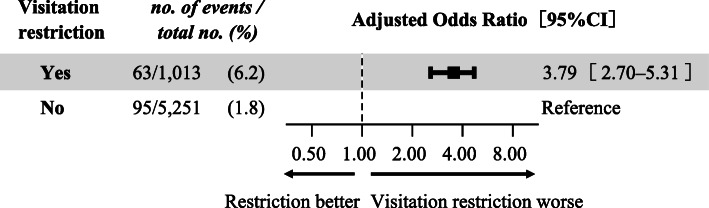
Forest plots of adjusted odds ratio for delirium incidence of visitation restriction. The logistic model was used to adjust for seven variables: age, sex, ward type on admission, primary diagnosis, ventilator management, general anesthesia surgery, and dementia

As the primary analysis, multivariable logistic regression analysis showed that the AOR for incidence of delirium after visitation restriction was 3.79 (95% CI, 2.70–5.31) compared with before visitation restriction. Forest plots of the AOR for delirium incidence after visitation restriction are shown in Fig. 3. The AORs of other confounders are shown in Supplementary Table 3.

### Subgroup analysis

**Fig. 4:**
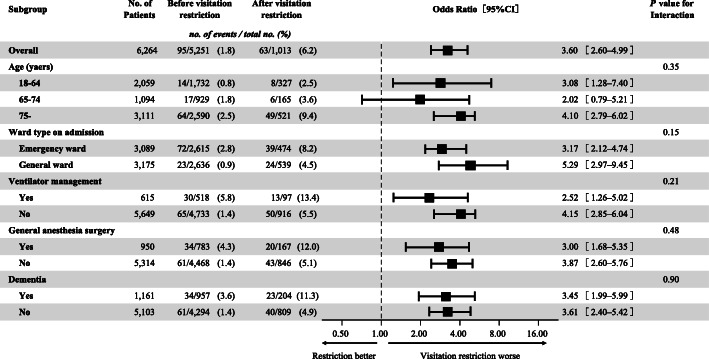
Forest plots of the subgroup analysis for the impact of visitation restriction on the incidence of delirium

Subgroup analysis showed that no apparent interaction for delirium incidence was observed regardless of age, ward type on admission, ventilator management, general anesthesia surgery, or dementia (Fig. 4). The *P* values for interaction were not significantly different in any of the analyses. Subgroup analysis based on the primary diagnosis was not attempted because each subgroup was too small and the number of subgroup cases was too small.

### Sensitivity analysis

The AOR for the incidence of delirium after visitation restriction was 3.71 (95% CI, 2.65–5.20). “The assumed SOFA score” as the patient severity of illness was 1.09 (95% CI, 1.02–1.17). The AORs of other confounders are shown in Supplementary Table 4. The results of this sensitivity analysis were nearly consistent compared with those of the primary analysis, even after adjusting for patient severity; thus, we believe that the results of the primary analysis were robust.

## Discussion

### Key observation

This retrospective single-center observational study showed an association between visitation restriction during the COVID-19 pandemic and the incidence of delirium among emergency inpatients. Furthermore, subgroup analysis indicated that the visitation restriction affect the delirium incidence regardless of the age, ward type on admission, ventilator management, general anesthesia surgery, and dementia.

### Strengths of the study

One of the strengths of this study was that it was the first to show that total visitation restriction was associated with the incidence of delirium in emergency inpatients during visitation restriction. Although some studies have focused on the association between partial limitation of visitation time and delirium in the intensive care unit setting [29–31], an assessment of the intervention of uniform and complete visitation restriction for emergency inpatients have never been performed. Because it is practically impossible to this type of study perform from an ethical standpoint, the association between total visitation restriction and the incidence of delirium has not been examined. During the intervention period for this present study, the entire world has faced the unprecedented disaster of the COVID-19 pandemic, and in response, medical facilities have had to implement the long-term visitation restrictions that have never before been implemented. In this context, the present study could investigate and show an association between complete visitation restriction and the incidence of delirium, which otherwise could not have been examined and is a unique strength of this study.

The second strength of this study is that the results are valid because the design is considered to be a kind of *natural experiment*, defined as a way to assess the impact of the intervention or changing policy for which planned and controlled experimental research designs may be infeasible or inappropriate to implement [32, 33]. Similar to a randomized controlled trial, the approach to this study has a strength in that the patient’s background and treatment can be considered equipoise before and after the intervention and that the effects of unmeasured confounding may be less pronounced. Therefore, this study design has recently attracted interest as an alternative to randomized controlled trial [33]. Although a randomized trial on visitation restrictions is neither practically nor ethically feasible to conduct, in the case of this current study, the intervention of the visitation restriction could be evaluated “as if” it was an experiment, albeit not under control. Therefore, the presence or absence of visitation restriction was considered to be the only variable in the two cohorts, and the effect of unmeasured confounding was likely to be small. In this regards, the results of this study would be highly valid, and thus this validity appears to be a strength.

### Interpretation of the results

To interpret the results of this study, the following potential mechanisms are suggested: visitation restrictions for families and close contacts may increase patients’ loneliness and anxiety in an unfamiliar hospital setting, thereby reducing cognitive stimulation such as conversation and engagement with the external world, and these factors may contribute to incidence of delirium. In general, hospitalization alters the functioning in the patients’ family by threatening familial roles and communication and by creating stress, anxiety, and discomfort for patients and families [34, 35]. Patients express their discomfort as denial, hostility, and anger [35]. Visitation by their family members can decrease their discomfort with the daily routine of the hospital [36]. Visitation also promotes stress relief, mental peace, and patient rest and reduces patient anxiety, anger, and hostility [34, 37]. Moreover, the presence of family in a critical care setting has been suggested as a means to achieve better pain control, to reduce the use of sedatives, and to contribute to the reorientation and cognitive stimulation for patients [31]. These benefits have been associated with lower incidence of delirium in studies evaluating multicomponent nonpharmacologic interventions to prevent delirium; these guidelines emphasized the importance of family involvement [15, 16, 31]. Family members also desire to be physically present with the patient to enhance their emotional support [36, 37]. Therefore, the authors of this current study believe that the visitation restriction that interfered with this effective family support may have increased the incidence of delirium for patients.

Furthermore, the authors suggest that the effect of the visit restriction on the delirium incidence may be universal, regardless of the individual patient characteristics. Subgroup analysis showed that visitation restriction affected the delirium incidence regardless of the major risk factors for delirium reported in previous literatures, such as patient age, ward type on admission, ventilator management, general anesthesia surgery, and dementia [1, 20–26]. We hypothesized that the degree of visitation restriction might play an important role in the incidence of delirium. The most recent randomized controlled study on the effect of family visits on delirium incidence demonstrated that there was no difference in delirium incidence with different family visitation policies [29]. This randomized controlled study compared time-limited (standard restricted visitation) and flexible visitation (up to 12 h/day). As mentioned earlier, previous studies have focused on the association between the partial limitation of visitation time and delirium [29–31]. In contrast, the present study focused on the association between complete visitation restriction and the incidence of delirium in emergency inpatients. Regarding the difference between previous studies and our study, the study populations might have played an important role; however, from our subgroup analysis, visitation restriction was associated with delirium incidence irrespective of the patient age. Based on this foundation, the effect of being forced into an extraordinary life during hospitalization, in which the patient is unable to meet family because of complete visitation restrictions, may be an emotional burden for all type of patients.

### Clinical and research implications

The authors believe that these results can be applied to clinical practice through dual efforts to recognize the association between visitation restriction and delirium incidence and to prevent delirium. Even patients who do not require emergency surgery or ventilator management, such as those admitted to general wards, should be alerted to the incidence of delirium during visitation restriction; therefore, it is necessary for clinicians and hospital staff to make a conscious effort to prevent delirium by talking to the patient and providing other stimuli. The COVID-19 pandemic may continue well into the future. This study highlighted that clinicians should not solely focus on COVID-19 infection control alone but also on the overall care of hospitalized patients, which may have been neglected in the face of the pandemic. If it is not possible to alleviate visitation restrictions, then the authors believe that measures such as online remote visitation systems to replace the traditional hospital visitation may be necessary. Moreover, the following research implications are suggested. Although this study focused on emergency inpatients, it is natural for the question to arise about the association between visitation restriction and delirium among the patients who have a planned admission and the difference between emergency versus planned admissions. One hypothesis is that the impact of visitation restriction may be stronger for emergency patients because of the sudden hospitalization in contrast to planned admissions. According to these considerations, further research is needed for the association between visitation restrictions and the incidence of delirium in hospitalized patients with planned admissions and for the effects of online or remote visitation systems.

### Limitations

This study has several limitations. First, this study may have an outcome detection bias. In our routine clinical practice, patients emergently admitted to our hospital were evaluated for the risks of delirium, such as age or activity of daily living, and the clinicians carefully managed them based on this risk screening. However, there were no objective criteria to consult the psychiatry department. Therefore, the decision for consultation by individual chief physicians may have affected the frequency of psychiatric consultations and the number of delirium diagnoses. Because of the retrospective nature of this study, the total number of psychiatry consultations was not obtained, and we could not deal with these risks of detection bias. In accordance, it may be necessary to interpret the results with caution.

Second, the study did not consider the severity or duration of the delirium. The duration and extent of delirium should have been considered; however, accurate data for these points was not available from medical record review. Third, some potential unmeasured confounders may influence the results. Fourth, regarding the single-center study design, the generalizability of these results to another hospital is unclear. Further study will be necessary to eliminate these potential biases.

## Conclusions

This study showed that visitation restriction was associated with an increased incidence of delirium for emergency inpatients.

## Supplementary Information

**Additional file 1: Appendix 1.** Explanation of sensitivity analysis. **Table S1.** The assumed Sequential Organ Failure Assessment (SOFA) score. **Table S2.** Detailed primary diagnosis for patients before and after visitation restriction. **Table S3.** Multiple logistic regression analysis for the incidence of delirium. **Table S4.** Additional sensitivity analysis: multiple logistic regression analysis for the incidence of delirium, included “the assumed SOFA score” as a confounder.

## Data Availability

Not applicable.

## Abbreviations

AOR: Adjusted odds ratio
CI: Confidence interval
COVID-19: Coronavirus disease
DPC: Diagnosis procedure combination
IQR: Interquartile range
SOFA: Sequential Organ Failure Assessment
