# Supplementary material for: Association between visitation restriction during the COVID-19 pandemic and delirium incidence among emergency admission patients: a single-center retrospective observational cohort study in Japan
Source: J Intensive Care. 2020 Dec 7;8:90. doi: 10.1186/s40560-020-00511-x (PMC7719735; doi:10.1186/s40560-020-00511-x)
Supplement: Supplementary file 1 — Additional file 1: Appendix 1. Explanation of sensitivity analysis. Table S1. The assumed Sequential Organ Failure Assessment (SOFA) score. Table S2. Detailed primary diagnosis for patients before and after visitation restriction. Table S3. Multiple logistic regression analysis for the incidence of delirium. Table S4. Additional sensitivity analysis: multiple logistic regression analysis for the incidence of delirium, included “the assumed SOFA score” as a confounder. [file 40560_2020_511_MOESM1_ESM.docx]

**Additional file**

**Title:** Association between visitation restriction during the COVID-19 pandemic and delirium incidence among emergency admission patients: A single-center retrospective observational cohort study in Japan

**Authors**

Kenji Kandori^1^, Yohei Okada^2,3^, Wataru Ishii^1^, Hiromichi Narumiya^1^, Yoshiro Maebayashi^4^, Ryoji Iizuka^1^

**Contents**

| **Supplementary Appendix 1.** | **Explanation of sensitivity analysis** |
| --- | --- |
| **Supplementary Table 1.** | **The assumed Sequential Organ Failure Assessment (SOFA) score** |
| **Supplementary Table 2.** | **Detailed primary diagnosis for patients before and after visitation restriction** |
| **Supplementary Table 3.** | **Multiple logistic regression analysis for the incidence of delirium** |
| **Supplementary Table 4.** | **Additional sensitivity analysis: multiple logistic regression analysis for the incidence of delirium, included “the assumed SOFA score” as a confounder** |

**Supplementary Appendix 1. Explanation of sensitivity analysis**

We performed a sensitivity analysis that also adjusted for physiological severity as a covariate to demonstrate the robustness of the main analysis, because the patient physiological severity might also be a potential confounder.

**“The assumed SOFA score”**

Generally, physiological severity is assessed by the Sequential Organ Failure Assessment (SOFA) score. Unfortunately, we could not obtain the SOFA score for all the patients and the following information; Glasgow coma scale, the respiratory condition, total bilirubin, serum creatinine, platelet count, and the detail of catecholamine dosage which are necessary to calculate SOFA score. Therefore, we alternatively assessed physiological status as “the assumed SOFA score” (**Supplementary Table 1**). For level of consciousness, because we could not collect Glasgow coma scale for all the patients; thus, we alternatively chose the Japan coma scale (JCS) which are measured and collected for all the admitted patients due to the Japanese Diagnosis Procedure Combination (DPC) system, instead of Glasgow coma scale. Based on the previous studies [1-3], we fit the JCS scale to the consciousness category in SOFA score. Further, we alternatively calculate the “assumed SOFA score” using following factors; ventilation management (Yes/No) as respiratory condition, catecholamine usage (Yes/No) as hemodynamic state, renal replacement therapy(Yes/No) as renal function and plasma exchange (Yes/No) as liver function during the hospital stay.

**Variables selection**

As potential confounding factors to assess the association between incidence of delirium and visitation restriction, the sensitivity analysis used these seven variables: patient age, patient sex, ward type on admission, primary diagnosis, general anesthesia surgery, dementia, and “the assumed SOFA score.”

**Statistical analysis**

As sensitivity analysis, the adjusted odds ratios (AORs) of delirium incidence with 95% confidence intervals (CIs) were identified using the multivariable logistic model including seven confounders as mentioned above.

**References**

1. Shigematsu K, Nakano H, Watanabe Y. The eye response test alone is sufficient to predict stroke outcome--reintroduction of Japan Coma Scale: a cohort study. BMJ Open. 2013;3:e002736.

2. Kurogi R, Kada A, Nishimura K, Kamitani S, Nishimura A, Sayama T, et al. Effect of treatment modality on in-hospital outcome in patients with subarachnoid hemorrhage: a nationwide study in Japan (J-ASPECT Study). J Neurosurg. 2018;128:1318-26.

3. Okada Y, Kiguchi T, Iiduka R, Ishii W, Iwami T, Koike K. Association between the Japan Coma Scale scores at the scene of injury and in-hospital outcomes in trauma patients: an analysis from the nationwide trauma database in Japan. BMJ Open. 2019;9:e029706.

**Supplementary Table 1. The assumed Sequential Organ Failure Assessment (SOFA) score**

|  |  |  | **Score** | | | | |
| --- | --- | --- | --- | --- | --- | --- | --- |
|  | **Variables** | | **0** | **1** | **2** | **3** | 4 |
|  | **Central nervous system** | |  |  |  |  |  |
|  |  | Japan Coma Scale | 0 | 1–20 | 30 | 100–300 |  |
|  | **Respiratory** | |  |  |  |  |  |
|  |  | Ventilator management | No |  |  | Yes |  |
|  | **Cardiovascular** | |  |  |  |  |  |
|  |  | Catecholamine usage | No |  |  | Yes  (any dose) |  |
|  | **Liver** | |  |  |  |  |  |
|  |  | Plasma Exchange | No |  |  | Yes |  |
|  | **Renal** | |  |  |  |  |  |
|  |  | Renal Replacement Therapy | No |  |  | Yes |  |
|  | **Coagulation** | |  |  |  |  |  |
|  |  |  | All patients |  |  |  |  |

SOFA: Sequential Organ Failure Assessment score

**Supplementary Table 2. Detailed primary diagnosis for patients before and after visitation restriction**

| **Variables, number, (% or IQR)** | | **All patients** | **Visitation restriction** | |
| --- | --- | --- | --- | --- |
|  |  |  | **Before (2019.01–2020.03)** | **After (2020.04–2020.06)** |
|  |  | **(N = 6264)** | **(N = 5251)** | **(N = 1013)** |
| **Primary diagnosis, n** | |  |  |  |
|  | **cerebral infarction** | 638 (10.2%) | 523 (10%) | 115 (11.4%) |
|  | **cerebral hemorrhage** | 243 (3.9%) | 204 (3.9%) | 39 (3.8%) |
|  | **subarachnoid hemorrhage** | 71 (1.1%) | 60 (1.1%) | 11 (1.1%) |
|  | **epilepsy** | 153 (2.4%) | 124 (2.4%) | 29 (2.9%) |
|  | **acute coronary syndrome** | 193 (3.1%) | 166 (3.2%) | 27 (2.7%) |
|  | **heart failure** | 216 (3.4%) | 186 (3.5%) | 30 (3.0%) |
|  | **pneumonia** | 240 (3.8%) | 201 (3.8%) | 39 (3.8%) |
|  | **upper gastrointestinal bleeding** | 89 (1.4%) | 78 (1.5%) | 11 (1.1%) |
|  | **adhesive intestinal obstruction** | 111 (1.8%) | 94 (1.8%) | 17 (1.7%) |
|  | **cholecystitis** | 61 (1.0%) | 49 (0.9%) | 12 (1.2%) |
|  | **cholangitis** | 131 (2.1%) | 112 (2.1%) | 19 (1.9%) |
|  | **appendicitis** | 102 (1.6%) | 74 (1.4%) | 28 (2.8%) |
|  | **urinary tract infection** | 126 (2.0%) | 97 (1.8%) | 29 (2.9%) |
|  | **pregnancy, childbirth, puerperium** | 344 (5.5%) | 305 (5.8%) | 39 (3.8%) |
|  | **sepsis** | 77 (1.2%) | 63 (1.2%) | 14 (1.4%) |
|  | **malignancy** | 211 (3.4%) | 193 (3.7%) | 18 (1.8%) |
|  | **trauma** | 979 (15.6%) | 826 (15.7%) | 153 (15.1%) |
|  | **COVID-19 infection** | 10 (0.2%) | 0 (0.0%) | 10 (1.0%) |

Values are median (interquartile range [IQR]) or number (percentage).

**Supplementary Table 3.** **Multiple logistic regression analysis for the incidence of delirium**

| **Variables** | | **n/N** | **(%)** | **Crude OR [95%CI]** | **Adjusted OR [95%CI]** |
| --- | --- | --- | --- | --- | --- |
| **Age** | |  |  |  |  |
|  | **65-74 years** | 23/1094 | (2.10) | 1.99 [1.10–3.58] | 1.64 [0.89–3.02] |
|  | **75- years** | 113/3111 | (3.63) | 3.49 [2.20–5.53] | 3.12 [1.90–5.13] |
| **Male** | | 95/3303 | (2.88) | 1.36 [0.99–1.88] | 1.26 [0.90–1.78] |
| **Primary diagnosis** | |  |  |  |  |
|  | **Neurological disease** | 24/1243 | (1.93) | 0.67 [0.38–1.17] | 0.40 [0.22–0.73] |
|  | **Cardiovascular disease** | 25/794 | (31.5) | 1.10 [0.63–1.92] | 0.72 [0.40–1.28] |
|  | **Respiratory disease** | 23/580 | (3.97) | 1.40 [0.80–2.47] | 1.37 [0.76–2.47] |
|  | **Digestive disease** | 17/1249 | (1.36) | 0.47 [0.25–0.86] | 0.50 [0.26–0.95] |
|  | **Pregnancy, gynecological disease** | 1/413 | (0.24) | 0.08 [0.01–0.61] | 0.44 [0.06–3.52] |
|  | **Trauma** | 41/1042 | (3.93) | 1.39 [0.85–2.28] | 1.00 [0.59–1.72] |
|  | **Others** | 27/943 | (2.86) | Reference | Reference |
| **Visitation restriction** | | 63/1013 | (6.22) | 3.60 [2.60–4.99] | 3.79 [2.70–5.31] |
| **Emergency ward on admission** | | 111/3089 | (3.59) | 2.48 [1.76–3.50] | 2.15 [1.45–3.20] |
| **Ventilator management** | | 43/615 | (6.99) | 3.62 [2.52–5.19] | 2.32 [1.53–3.52] |
| **General anesthesia surgery** | | 54/950 | (5.68) | 3.02 [2.16–4.23] | 2.95 [2.01–4.33] |
| **Dementia** | | 57/1161 | (4.91) | 2.56 [1.84–3.56] | 2.07 [1.45–2.96] |

AOR, adjusted odds ratio; CI, confidence interval; COR, crude odds ratio.

Adjusted by confounding variables including visitation restriction, age, sex, ward type on admission, primary diagnosis, ventilator management, general anesthesia surgery, and dementia.

**Supplementary Table 4. Additional sensitivity analysis: multiple logistic regression analysis for the incidence of delirium, included “the assumed SOFA score” as a confounder**

| **Variables** | | **n/N** | **(%)** | **Adjusted OR [95%CI]** |
| --- | --- | --- | --- | --- |
| **Age** | |  |  |  |
|  | **65-74 years** | 23/1094 | (2.10) | 1.63 [0.88–3.00] |
|  | **75- years** | 113/3111 | (3.63) | 3.05 [1.86–5.01] |
| **Male** | | 95/3303 | (2.88) | 1.27 [0.90–1.79] |
| **Primary diagnosis** | |  |  |  |
|  | **Neurological disease** | 24/1243 | (1.93) | 0.40 [0.22–0.72] |
|  | **Cardiovascular disease** | 25/794 | (31.5) | 0.75 [0.42–1.34] |
|  | **Respiratory disease** | 23/580 | (3.97) | 1.47 [0.81–2.68] |
|  | **Digestive disease** | 17/1249 | (1.36) | 0.50 [0.26–0.96] |
|  | **Pregnancy, gynecological disease** | 1/413 | (0.24) | 0.45 [0.06–3.56] |
|  | **Trauma** | 41/1042 | (3.93) | 1.00 [0.58–1.72] |
|  | **Others** | 27/943 | (2.86) | Reference |
| **Visitation restriction** | | 63/1013 | (6.22) | 3.71 [2.65–5.20] |
| **Emergency ward on admission** | | 111/3089 | (3.59) | 2.25 [1.51–3.35] |
| **General anesthesia surgery** | | 54/950 | (5.68) | 3.16 [2.16–4.63] |
| **Dementia** | | 57/1161 | (4.91) | 2.02 [1.41–2.90] |
| **The modified SOFA score/1point** | |  |  | 1.09 [1.02–1.17] |

AOR, adjusted odds ratio; CI, confidence interval; COR, crude odds ratio.

Adjusted by confounding variables including visitation restriction, age, sex, ward type on admission, primary diagnosis, general anesthesia surgery, dementia, and “the assumed SOFA score.”
